# The role of memory and perspective shifts in systematic biases during object location estimation

**DOI:** 10.3758/s13414-022-02445-y

**Published:** 2022-02-16

**Authors:** Vladislava Segen, Giorgio Colombo, Marios Avraamides, Timothy Slattery, Jan M. Wiener

**Affiliations:** 1 Aging and Dementia Research Centre, Bournemouth University, Poole, UK; 2 Department of Psychology, Bournemouth University, Poole, UK; 3 German Center for Neurodegenerative Diseases, Magdeburg, Germany; 4 ETH Zurich, Future Health Technologies, Singapore-ETH Centre, CREATE Tower, Singapore; 5 Department of Psychology, University of Cyprus, Nicosia, Cyprus; 6 CYENS Centre of Excellence, Nicosia, Cyprus

**Keywords:** Spatial memory, Spatial perspective taking, Perception of space, Spatial cognition, Object location memory

## Abstract

In the current study, we investigated whether the introduction of perspective shifts in a spatial memory task results in systematic biases in object location estimations. To do so, we asked participants to first encode the position of an object in a virtual room and then to report its position from memory or perception following a perspective shift. Overall, our results showed that participants made systematic errors in estimating object positions in the same direction as the perspective shift. Notably, this bias was present in both memory and perception conditions. We propose that the observed systematic bias was driven by difficulties in understanding the perspective shifts that led participants to use an egocentric representation of object positions as an anchor when estimating the object location following a perspective shift.

**Supplementary Information:**

The online version contains supplementary material available at 10.3758/s13414-022-02445-y.

## Introduction

An important aspect of spatial cognition is the ability to recognize and remember spatial locations across different viewpoints (Epstein, et al., 1999; Waller & Nadel, 2013). This ability allows us to orient in situations when we encounter familiar places from different perspectives (e.g., when approaching an intersection from a different direction than on our usual way or when entering our kitchen through the backdoor). Broadly, in order to recognize locations from different perspectives, one needs to bind objects/landmarks that define the place to their spatial locations (Postma et al., 2004). Once such a spatial representation of a place is formed, self-motion information can be used to update the representation to allow recognition from a different perspective (Bülthoff & Christou, 2000; Waller et al., 2002). However, if physical movement is absent, recognition across different perspectives can be achieved by forming a viewpoint-independent spatial representation or by mentally manipulating a viewpoint-dependent representation, a process known as spatial perspective taking (Holmes et al., 2018; King et al., 2002; Klencklen et al., 2012).

In laboratory experiments, spatial perspective taking is typically assessed with tasks where participants first encode an array of objects or environmental features from one perspective and are then asked to indicate whether the array has changed when presented from a different perspective (Diwadkar & McNamara, 1997; Hartley et al., 2007; Hilton et al., 2020; Montefinese et al., 2015; Muffato et al., 2019; Schmidt et al., 2007; Segen et al., 2021a, 2021b; Sulpizio et al., 2013). Most previous studies employing such paradigms focus on the ability to remember object locations rather than on assessing the precision of the underlying representations. However, spatial representations can greatly vary in terms of the precision with which they are encoded (Evensmoen et al., 2013). For example, you can remember that the car is parked at a particular area in a car park, or you can formulate a more precise representation in which you remember the row in which the car is parked and the relative position in this row (back, center, front).

In our previous work (Segen et al., 2021c), we designed a novel task to assess the precision of spatial representations. The task required participants to memorize the position of an object in a virtual room. At test, the scene would be presented from a different perspective, the object would be displaced to either the participants’ egocentric left or right, and participants needed to decide in which direction the object had moved. To evaluate the precision of the object location representations across different perspectives, we adopted a psychophysics approach and systematically manipulated the object displacement distances with the aim of identifying the distance at which participants would be able to reliably detect the direction of movement. Unexpectedly, we found a systematic bias that was associated with the combination of the directions of the perspective shift and object movement, which we termed the Reversed Congruency Effect. Specifically, when the direction of the perspective shift and the object movement were congruent (e.g., the object moved to the right and the perspective shift was to the right), participants consistently misjudged the direction of the object movement for small object displacement distances. The opposite pattern was found in trials where the direction of the perspective shift and the object movement were incongruent (i.e., the perspective shift was in the opposite direction to the object movement direction). In this case, participants correctly identified the displacement direction regardless of the distance by which the object has moved.

It is not clear what gives rise to the Reversed Congruency Effect. However, given that responses are influenced by the direction of the perspective shift, it is likely that the bias results from egocentric, rather than allocentric, influences on the object position estimates. Specifically, if participants rely solely on an allocentric representation in which the position of the object is encoded relative to other features in the environment, their own position and movement in the environment should not influence their responses and perspective shifts should not result in systematic biases (Ekstrom et al., 2014). Furthermore, a recent study showed that for small viewpoint changes (<45°), participants are more likely to rely on egocentric rather than allocentric representations when deciding whether object locations have changed following a perspective shift (Heywood-Everett et al., 2020). Based on this result, and since we used a small perspective shift (20°), we consider it possible that participants in our past study were also biased towards relying on an egocentric representation.

Yet reliance on egocentric representations alone does not explain the systematic bias in participants’ responses as a function of the perspective shift. Even if object positions are encoded in relation to the participants’ position in the environment, those representations could be updated in an unbiased way via mental transformations that support spatial perspective taking. Thus, we propose that the bias is driven by uncertainty regarding how the perspective shift would affect the position of the objects on the screen (Segen et al., 2021c). Due to this uncertainty, participants may bias their representation of the location that the object has previously occupied towards the egocentric self-to-object estimates derived during encoding (cf. Epley et al., 2004).

**Fig. 1:**
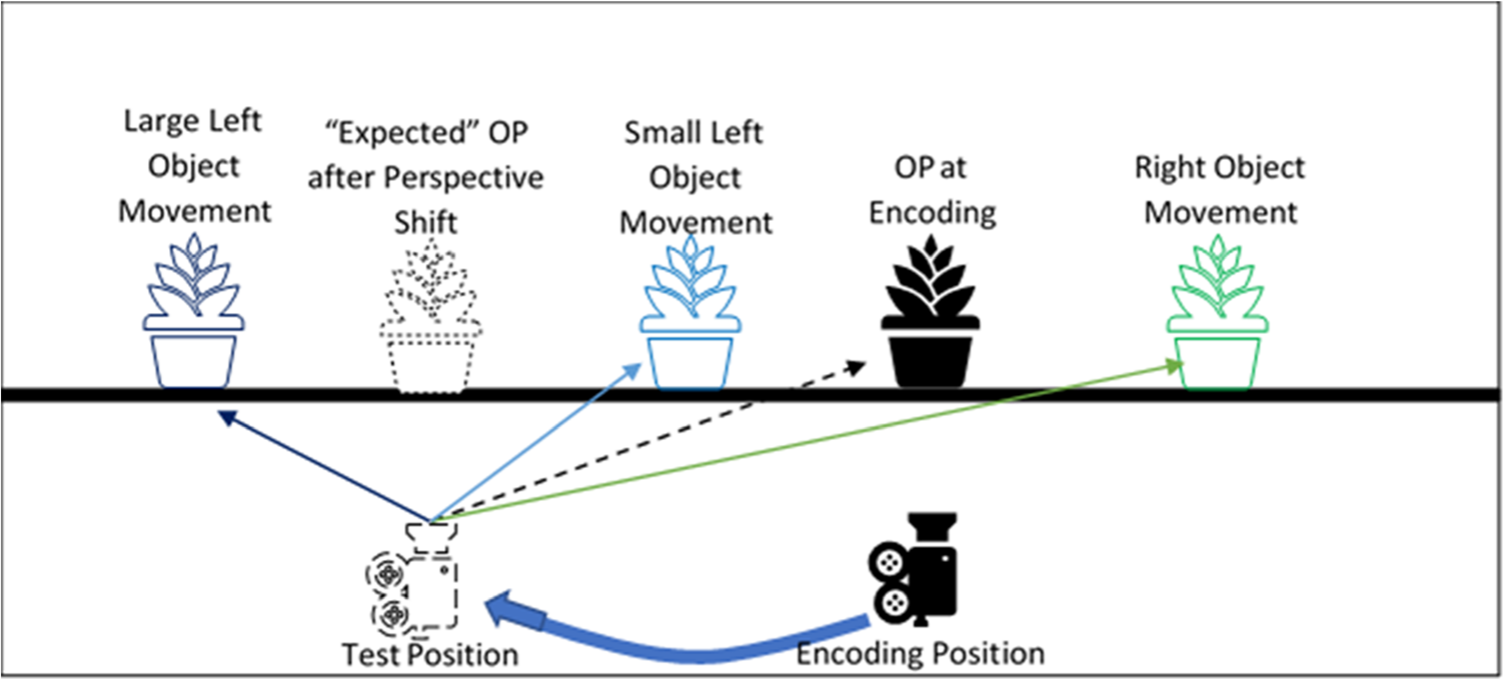
Schematic of the Reversed Congruency Effect: The black plant and camera represent the position of the object (OP) and camera at encoding. The dotted camera represents the position at test following a perspective shift to the left. The dotted plant represents the “expected” position of the object following a perspective shift if participants “drag” the object with them. Given the new position (dotted camera), it appears that even if the object remains stationary (black plant) that the object has moved right (i.e., perspective shift induced object motion). The green plant represents small movement to the right, which is perceived to be much larger due to the perspective shift induced object motion. Whilst small left movements (light-blue plant) are perceived as right movements due to being further to the right than the “expected” object position, yet, when the movements to the left (congruent with the direction of the perspective shift) were large enough (i.e., dark-blue plant), participants could correctly detect the movement direction. (Colour figure online)

This explanation suggests that participants “drag” the object in the same direction as the perspective shift. Thus, when the object remains stationary, participants “perceive” the object as having moved in the opposite direction of the perspective shift. Together with the actual object movement, this expectation that the object “moves” in the same direction as the perspective shift would yield the observed Reversed Congruency Effect. Specifically, if the object moved in the opposite direction to the perspective shift, participants would perceive the object movement to be larger due to the expectation that the object follows the perspective shift (Fig. 1). Whilst, in situations when the object moves in the same direction as the perspective shift, participants may incorrectly perceive the object movement direction, as the change in the object position may not be large enough to overcome their expectation regarding the new object position following a perspective shift (Fig. 1). Yet, in trials when object movement was large, the effect of the perspective shift-related expectation of object movement is overcome, allowing participants to correctly detect the direction in which the object moved (Fig. 1).

Although this explanation is in line with our empirical data, our original study (Segen et al., 2021c) did not allow us to directly investigate whether the Reversed Congruency Effect described above was primarily driven by the proposed perspective shift-related bias in which participants drag the object in the same direction as the perspective shift. Alternatively, it is possible that the Reversed Congruency Effect relied on the presence of the object in both the encoding and test phase and that the comparison of the object locations across those stimuli gave rise to the observed bias.

Thus, the primary aim of the current study was to investigate whether perspective shifts lead to a systematic bias in the remembered object positions. This question is particularly important as many studies investigating spatial memory and perspective taking abilities (Diwadkar & McNamara, 1997; Hartley et al., 2007; Hilton et al., 2020; Montefinese et al., 2015; Muffato et al., 2019; Schmidt et al., 2007; Segen et al., 2021a, 2021b; Sulpizio et al., 2013) rely on paradigms that entail the presentation of static images from different perspectives and could therefore be subject to a similar bias. Thus, providing a more nuanced understanding of how this bias comes about will not only inform the field of spatial cognition but will also help improve the design of future studies on spatial perspective taking.

To pursue this aim, we designed a task in which participants first encoded the position of an object. Then, they were presented with an image of the same scene but from a different perspective but without the object and had to indicate the position of the object. If, as argued above, the Reversed Congruency Effect is driven by a perspective shift-related bias, we expect that participants will produce systematic errors in the same direction as the perspective shift. That is, if the perspective shift is to the left, participants would place the object further to the left of its actual position.

An additional aim of the study was to investigate whether the potential perspective shift-related bias is related to memory processes. It is well known that spatial memory is prone to a wide range of distortions. For example, when drawing sketch maps of environments from memory, participants often draw nonorthogonal junctions as 90° junctions and straighten the curved street segments (Wang & Schwering, 2009). In addition, distance estimates are influenced by the presence of physical or geographical borders (Uttal et al., 2010). Memories for object locations are also prone to systematic biases. That is, many studies have shown that object location estimates tend to “move” towards category prototypes (Crawford & Duffy, 2010; Holden et al., 2010; Huttenlocher et al., 1991; Huttenlocher et al., 1994). Specifically, when asked to memorize the location of a dot in a circle, participants divide the circle into quadrants and estimate the dot position closer to the center of each quadrant (Huttenlocher et al., 1991). Such biases are present beyond the spatial domain. For example, in the boundary extension phenomenon, people remember more of a scene than was originally present in the studied stimuli (Intraub & Bodamer, 1993). Together, these results suggest that the locus of the bias in object location estimates may be the maintenance stage of the memory.

Additionally, it is possible that spatial perspective-taking abilities may be differentially affected in situations when the to be manipulated representation is held in memory or is perceptual available to the participant. For example, Hartley et al. (2007) showed that reliance on spatial memory leads to greater difficulties in spatial perspective taking. The authors suggested that this can be explained by the need to manipulate the whole scene to achieve perspective taking if the representation is held in memory. In contrast, when participants can see the scenes from both perspectives simultaneously, it is possible to use piecemeal rotation of each element in the scene to ensure that the positions between the two scenes match. Following this explanation, we would expect that the perspective shift-related bias would only be apparent when memory is involved, where perspective taking itself may be more complex.

Thus, in the present study we investigated whether memory contributes to the predicted perspective shift-related bias in the object locations by creating two conditions. In the *memory* condition, participants first saw the image of a scene with the target object during encoding and, after a short delay, the second image showing the same scene from a different perspective but without the object. Their task was to indicate, on the second image, the position of the object. In the *perception* condition, participants performed the same task, but the two images were presented simultaneously on two adjoining computer screens. If memory contributes to the systematic bias introduced by the presence of a perspective shift, we expect a stronger bias in the *memory* condition than in the *perception* condition. However, if the effect is driven by the introduction of the perspective shift and is independent of memory, we expect similar results across the two conditions.

## Method

### Participants

Seventy-seven participants took part in the experiment (Mean age = 19.94 years, *SD* =2.35; age range: 18–32 years; 49 females and 28 males) with 39 participants completing the *memory* condition and 38 the *perception* condition. Participants were recruited through Bournemouth University’s participant recruitment system and received course credit for their participation. All participants gave their written informed consent in accordance with the Declaration of Helsinki (World Medical Association, 2013).

### Materials

#### Virtual environment

**Fig. 2:**
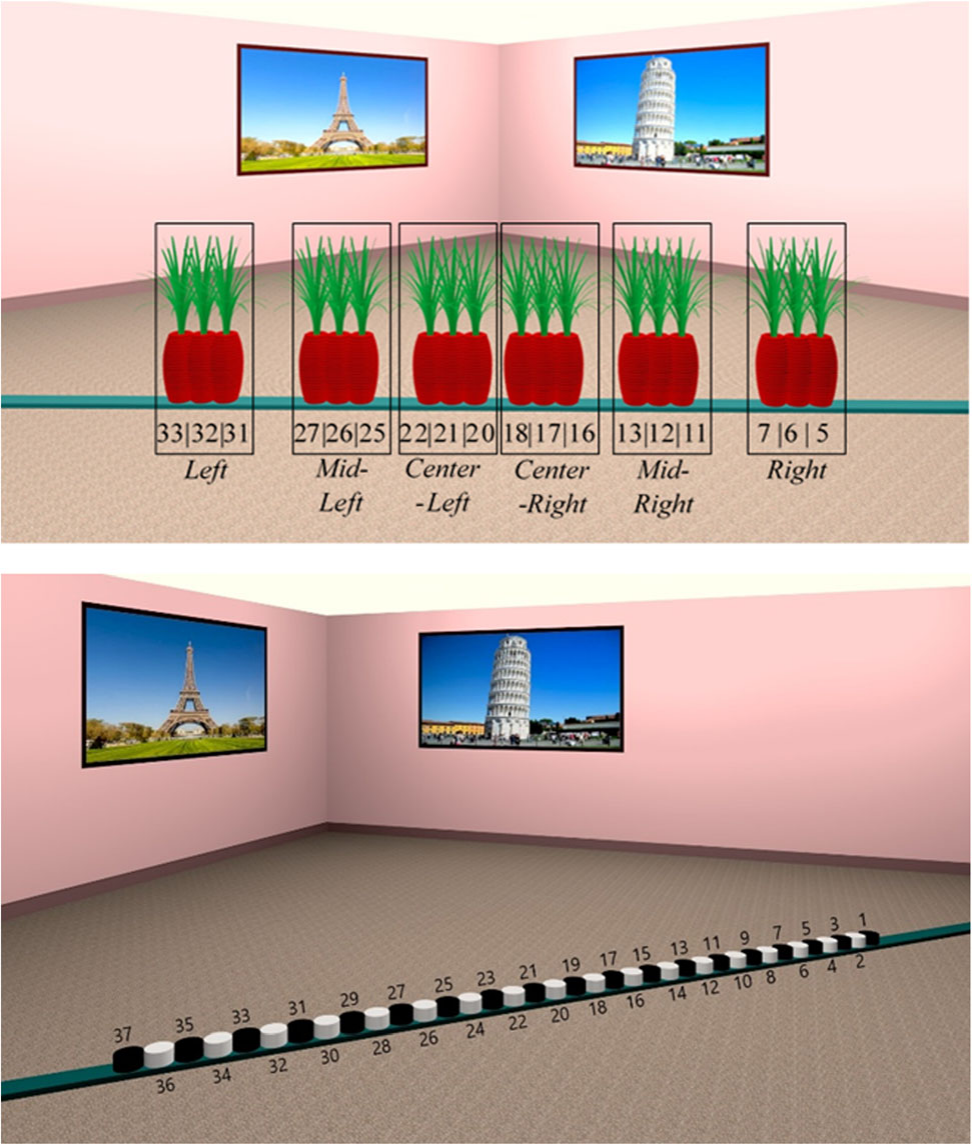
**a** Example stimuli superimposing all of the possible object positions ranging between 5 and 33 (positional markers in Fig. 2b) and the corresponding six object clusters (*left, mid-left, center-left, center-right, mid-right* and *right*). **b** Example of *test* stimuli containing the positional markers from 1 to 37 that participants needed to select to estimate object position (Colour figure online)

The virtual environment was designed with 3DS Max 2018 (Autodesk Inc) and consisted of a square room (9.8 m × 9.8 m) that contained famous and easily recognizable landmarks on its walls (Hamburger & Röser, 2014). A teal plank was placed diagonally in the middle of the room (14-m long). During encoding, an object was placed on that plank at one of 18 predefined positions that were 14, 28, 42, 84, 98, 112, 168, 182, and 192 cm to the left or to the right of the center of the plank. (Fig. 2a). The object was removed during testing, and 37 markers appeared on the plank serving as possible response locations (see Fig. 2b).

To analyze participant's responses, we created six groups containing three object positions (*left, mid-left, center-left, center-right, mid-right, right*) that were close to each other (i.e., objects positions at 14, 28, and 42 cm to the left of the center were grouped together; see Fig. 2a). From here on, we will refer to those object groups as object clusters.

**Fig. 3:**
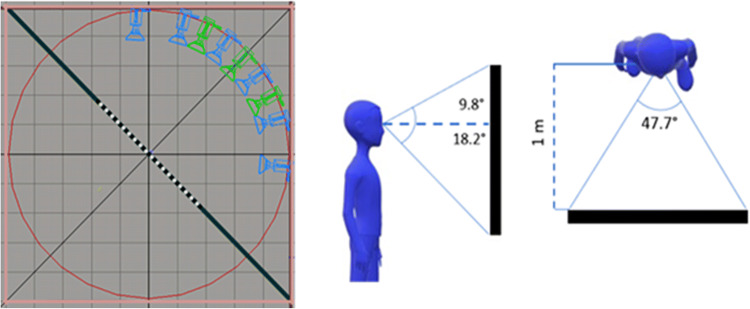
Left: schematic of encoding (green) and test (blue) camera positions arranged in an invisible circle in the environment. Right: A representation of how participant position related to the stimulus display. (Colour figure online)

The visual stimuli were presented on a 40-inch screen at a resolution of 1,920 × 1,080 px and subtended 47.7° × 28° at a viewing distance of 1 meter. The experimental stimuli were renderings of the environment with a 60° horizontal field of view (FOV), a custom asymmetric viewing frustum that resembles natural vision with a 15% shift in the vertical field of view was used (Franz, 2005; see Fig. 3).

The cameras were arranged in an invisible circle around an invisible diagonal line that was perpendicular to the plank. The encoding stimuli were rendered from three possible camera positions (see Fig. 3). The test stimuli were rendered from a different viewpoint with a 30° perspective shift either to the *left* or to the *right* of the encoding viewpoint. In both encoding and test stimuli, the room corner and one poster at each side of the corner were visible.

**Fig. 4:**
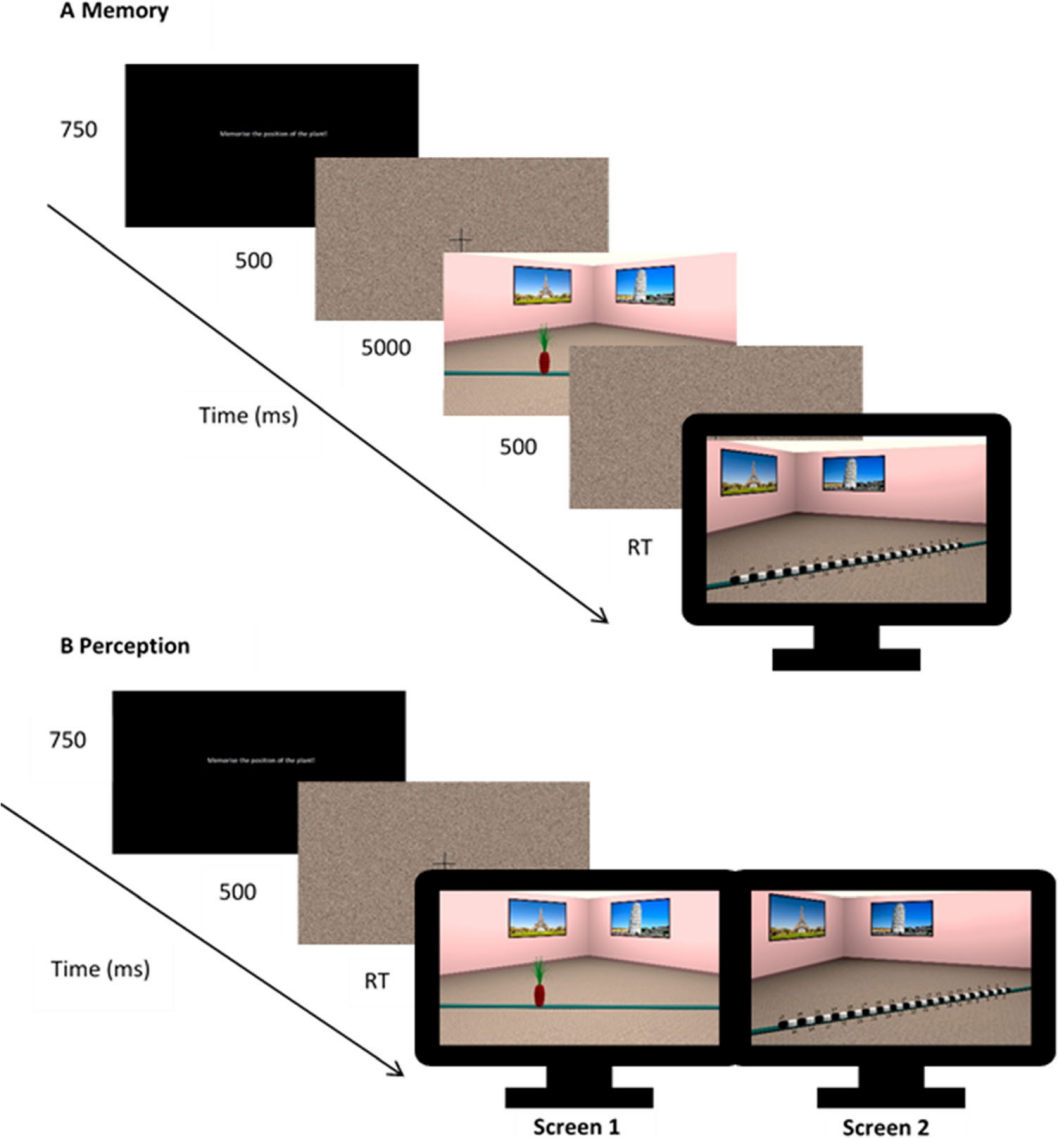
Trial structure in the *memory* (**a**) and *perception* (**b**) conditions (Colour figure online)

Stimuli were presented with OpenSesame 3.1.7 (Mathôt et al., 2012). In the *memory* condition, the stimuli were presented on a single monitor and in the *perception* condition stimuli were presented across two monitors (Fig. 4). Responses were made with a standard keyboard that was labelled such that a different key corresponded to each of the 37 possible positional markers. Participants had to choose the marker that they thought corresponded to the position of the object during encoding, and to press the key that corresponded to that marker (see Fig. 2b).

### Procedure

Each experimental trial started with the presentation of an instruction prompting participants to remember the location of the object (750 ms). This was followed by a display containing a fixation cross and a scrambled stimuli mask (500 ms). In the *memory* condition, this was followed by the encoding phase, in which participants were presented for 5 seconds with an image of the scene that depicted the object in one of the 18 possible positions in the room, taken from one of three camera positions. After the encoding phase, participants were again presented with a fixation cross and a scrambled stimuli mask for 500 ms. In the test phase that followed, they were presented with another image that was taken after a 30° perspective shift either to the *left* or to the *right*. In this image, the object was removed, and 37 labelled markers appeared on the plank which participants used to indicate object locations (see Fig. 4a). In the *perception* condition, participants were presented with the encoding and test stimuli simultaneously across two screens (Fig. 4b). In both conditions, participants were free to take as long as they needed to make a response.

### Design

A mixed design was adopted, and block randomization was used to assign participants to the *memory* or *perception* condition. This ensured an approximately equal number of participants in each condition. Perspective shift direction (*left*/*right*) was manipulated within participants. Overall, the experiment included 108 experimental trials presented in randomized order, with the experiment taking on average about 30 minutes.

### Data analysis

Data were analyzed with linear mixed-effects models (LME) using LME4 (Bates et al., 2015) in R (R Core Team, 2013). Effect coding was used as contrasts for fixed factors, which were all categorical variables. All of the LMM models included a by-item intercepts as well as a by-subject intercept. Prior to analysis, outlier responses were removed using the interquartile range method on individual absolute error (cm) distributions which led to a total 3.3% data loss. We present here analyses on signed and directional error. Analyses based on absolute error are presented in the supplementary material. The datasets used in the reported analyses are available in the Open Science Framework repository (https://osf.io/zkg2f/).

### Power analysis

We used the SIMR package (Green & MacLeod, 2016) in R to determine if our experiment was sufficiently powered to detect a difference between *memory* and *perception* conditions. Given that this was an exploratory study, there are no effect size estimates available in previous literature. As a result, the effect size (i.e., bias in object location estimates in the direction of the perspective shift in the *memory* condition) was chosen to be the minimum error that participants could make on a single trial (14 cm). SIMR power analysis revealed that 42 participants were needed to reach >80% statistical power to detect differences between the *memory* and *perception* conditions.^1^ Thus, given our sample size of 77 participants, we concluded that we had sufficient power to study the effect of interest.

## Results

### Signed error

Since we are primarily interested in the direction of the error as a function of perspective shift direction, we have focused our analysis on signed error. First, we estimated the magnitude of participants’ errors, by calculating the distance on the horizontal plane between the correct position and the position selected by the participant. Given the predefined arrangement of positional markers that participants used to give a response the minimum error could be 14 cm (unless participants select the correct position) and maximum error depended on the position of the object during encoding. Next, we estimated the direction of the error, such that errors to the left of the correct object position had a negative sign (i.e., −28cm) and errors to the right of the correct object position had a positive sign (i.e., 28 cm). Finally, we multiplied (folded) all of the errors where the perspective shift direction was to the *left* by −1. Following this folding procedure, positive errors indicate errors in the direction congruent with the perspective shift direction (i.e., camera moves to the left and participants make errors to the left of the correct object position) and negative errors indicate incongruent errors (i.e., camera moves *left* and participants make errors to the right of the correct object position), thereby allowing us to investigate the direction of the errors as a function of the perspective shift direction.

**Fig. 5:**
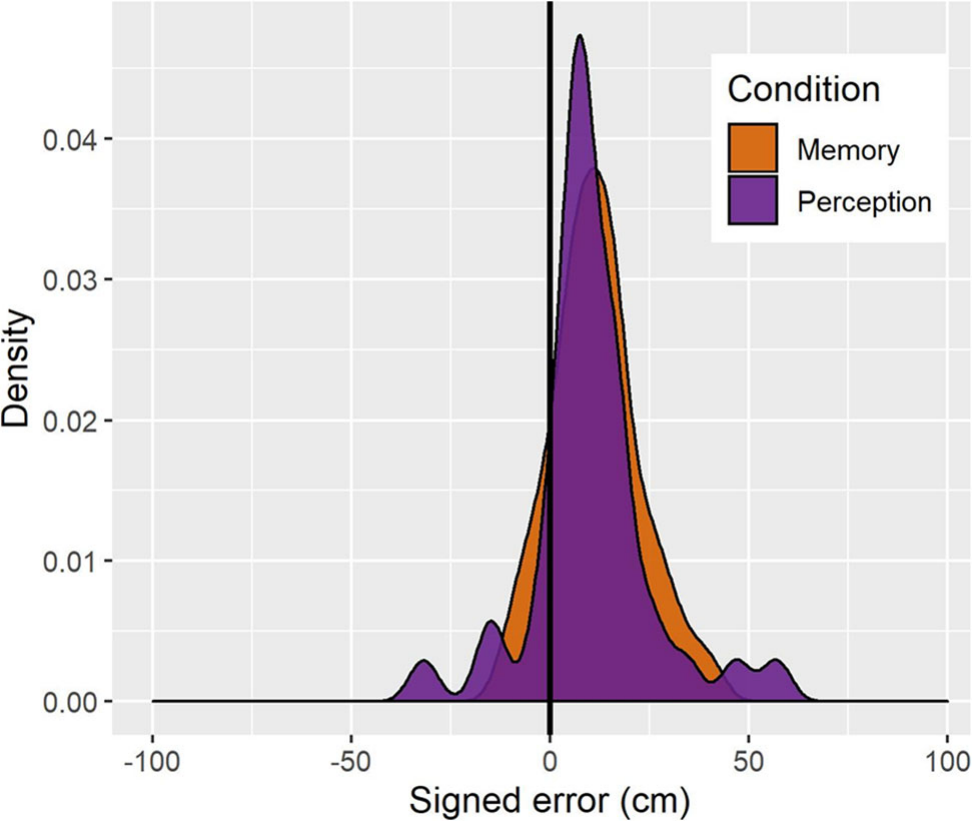
Density plot of signed error (cm) across the *memory* and *perception* conditions (Colour figure online)

An LMM with Condition as a fixed effect revealed that overall, the signed error was positive (Intercept: β = 11.077, *SE* = 2.012, *t* = 5.505). In other words, responses were biased towards the direction of the perspective shift (Fig. 5). Signed error did not differ between the *memory* and the *perception* conditions (β = −0.555, *SE* = 1.534, *t* = −0.362).

### Role of object position

Given previous reports of systematic biases in object location memory (Huttenlocher et al., 1991) towards a “category” prototype, we examined if object positions had an impact on participants’ errors. To do so we calculated, using the response markers, the range of responses for each of the 18 object positions, such that the value of 0 corresponds to responses in which the participants placed the object in the correct position, negative values represent errors made to the left, and positive values indicate errors to the right. Figure 7 displays histograms of responses for each object position. To investigate if participants' responses for each object position were significantly different from zero, thus indicating a systematic bias, we ran one-sample *t* tests for each object position separately for the *memory* and *perception* conditions.

**Fig. 6:**
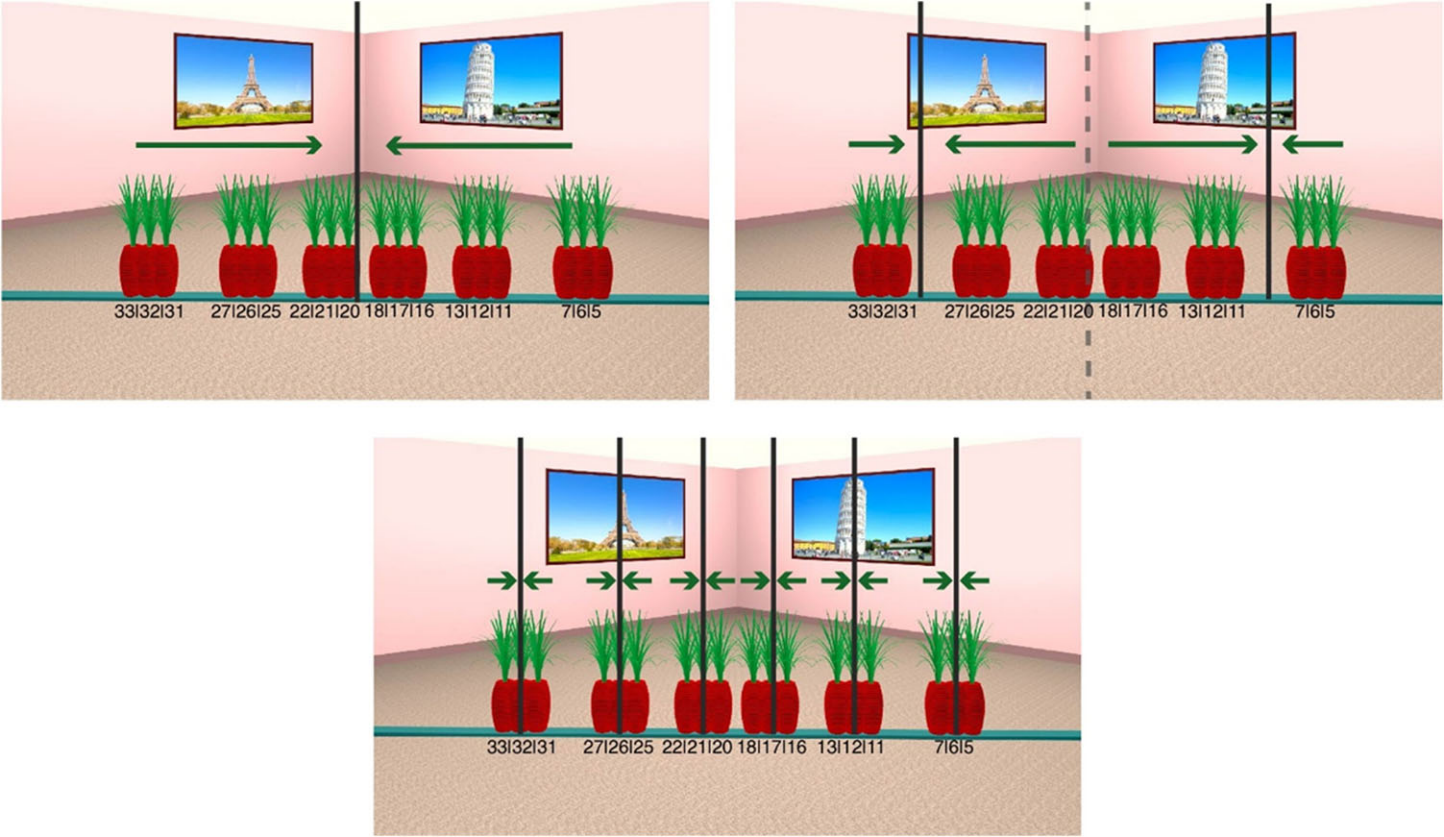
Examples of possible object position prototypes that participants may use, with the green arrows indicating the expected bias direction. Black lines indicate prototype locations. Center of the screen (**a**), center of the left and right side of the screen (**b**), or center of the cluster (**c**) used as a category prototype (Colour figure online)

As it is not clear what prototypes participants might have used in the current task, we evaluated different alternatives suggested by the previous literature. For example, one possibility is that participants remembered objects to be closer to the center of the screen (conceptually similar to the central tendency bias; e.g., Allred et al., 2016; see Fig. 6a). If participants indeed used the center of the screen as the prototypical object position, we would expect them to make errors to the left for object positions 5 to 18, and to the right for object positions 20 to 33 (Fig. 6a). Another possibility is that participants divided the plank into two halves and used the center of each half as prototypical locations (Crawford & Duffy, 2010; Huttenlocher et al., 1994). If participants used the center of those halves as prototypes, we would expect a leftward bias in object positions 5 to 7 and a rightward bias for object positions 11 to 18, as this would bring objects positioned on the right closer to the center of the right half of the plank. For the left half of the stimuli, we would expect a leftward bias for object positions 20 to 27 and a rightward bias for object positions 31 to 33 (Fig. 6b). Another possibility is that participants used more fine-grained categories in which the object in the center of each of the six object clusters functioned as a category prototype (see Fig. 6c; Holden et al., 2010). This way, in the cluster consisting of object positions 31, 32, and 33, participants would estimate the object positions to be closer to object position 32.

**Fig. 7:**
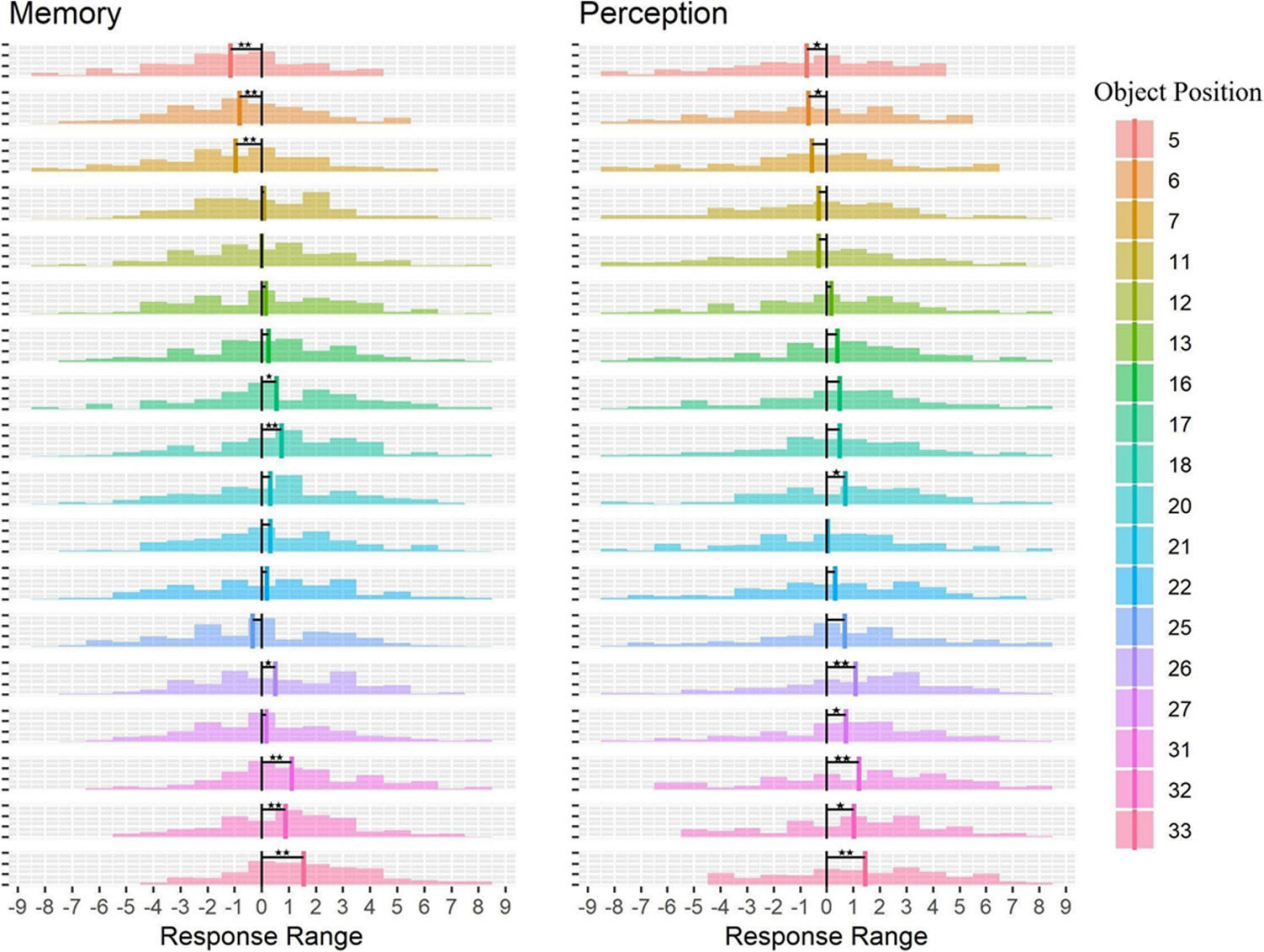
Distribution of the response range for each object position as a function of condition (*memory* and *perception*) (Colour figure online)

Our results showed that for objects positioned at the extremes of the possible object positions (most leftward [i.e., 33, 32, 31] and most rightward [5, 6, 7] positions), participants made errors away from the extreme values (the positional markers on both ends; Fig. 7). For example, for object positions 33 and 32, which are on the left side of the plank, participants made more errors to the right, whilst for object positions 5, 6, and 7 that are on the right, participants made more errors to the left. This result is partly in line with the category prototypes depicted in Fig. 6a–b. However, for the more central object positions, we found a slight bias to the right that is not consistent with any of the possibilities we described (Fig. 6).

**Fig. 8:**
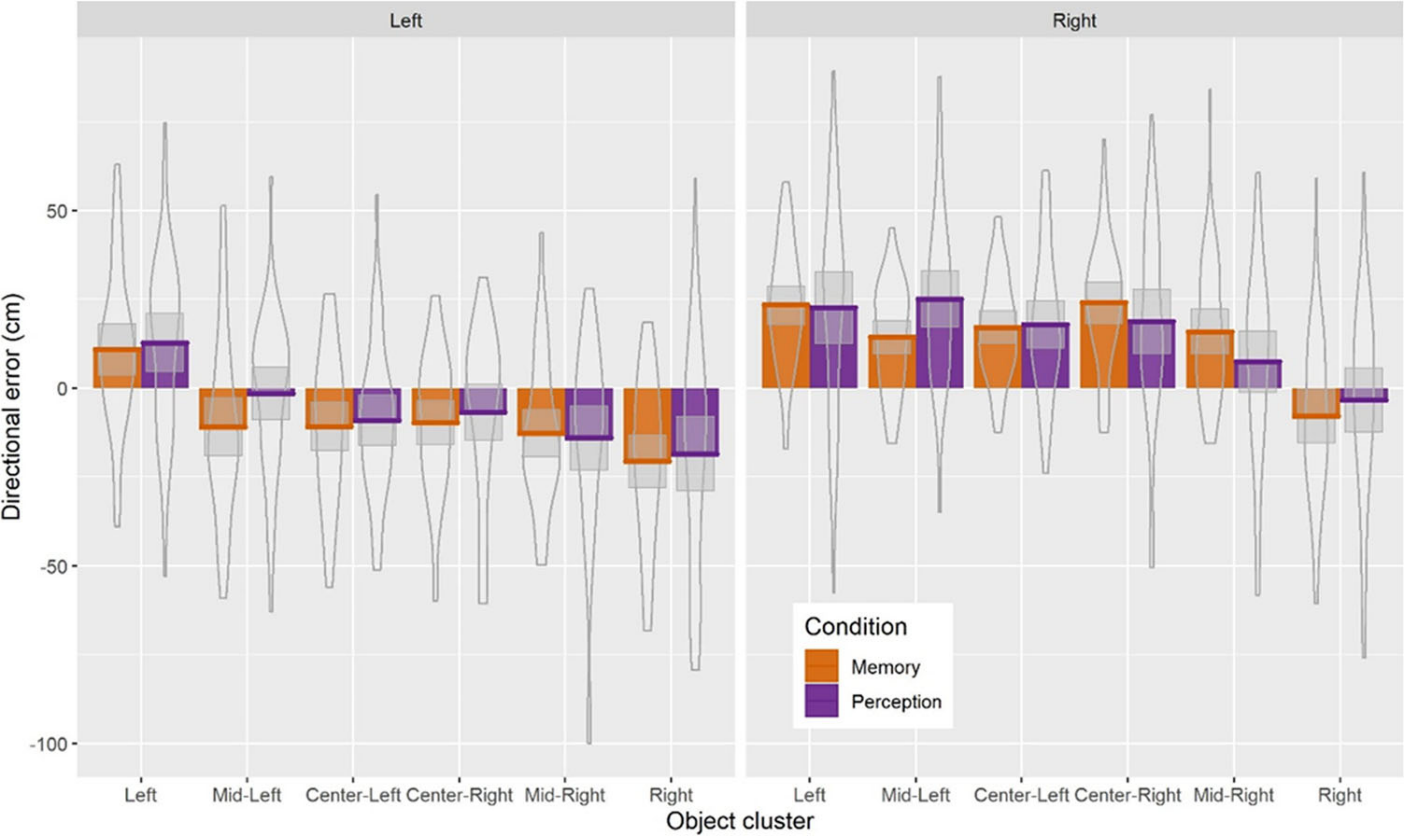
Bar plots for directional error as a function of perspective shift direction, condition, and object cluster with mean (solid line) and 95% CIs (grey-shaded area) with violin plots behind (Colour figure online)

We have also looked at directional errors (i.e., negative errors are errors to the left and positive errors are errors to the left of the correct object position) with the complete model reported in the Supplementary Materials. Overall, results are consistent with the signed error analysis that the direction of the perspective shift determined the direction of the errors. That is, when the perspective shift was to the *right* then the errors were to the right (positive errors). This was the case across all but the most leftward and rightward object clusters, for which we found that participants made errors away from the extremes such that the direction of the perspective shift no longer determined the direction of the errors. Instead, participants made more errors to the right in the left cluster, with the opposite pattern of errors found for the most rightward object cluster (Fig. 8). Given that the most leftward and rightward positioned objects elicited different response strategies in participants compared with the remaining object positions (Figs. 7 and 8), with a systematic shift away from the extremes (Figs. 7 and 8), we have re-run the signed and directional errors analysis without the left and right object clusters (results reported in supplementary material). Removal of those extreme positions in signed error analysis resulted in a larger bias in the direction of perspective shift direction (11.08 cm vs. 13.45 cm). Similarly, the effect of perspective shift direction (*left/right*) was strengthened in the directional error analysis, with all of the object clusters behaving in line with the perspective shift-related bias (i.e., object location estimates were biased in the direction of the perspective shift direction).

## Discussion

The main aim of this study was to investigate if perspective shifts systematically bias estimates for object positions. Consistent with our expectations, we found that participants’ estimates of object locations were systematically biased in the direction of the perspective shift, an effect we termed the perspective shift-related bias. Importantly, this perspective shift-related bias was observed in both the *memory* and *perception* conditions, suggesting that it is not related to systematic distortions in memory.

But how can this systematic perspective shift-related bias in object location estimation be explained? Our conjecture is that uncertainty about the exact nature of the perspective shift leads to uncertainty about the exact object location, which in turn results in participants biasing their estimates towards the encoded egocentric location of the object. This idea is conceptually similar to the anchoring and adjustment heuristic proposed by Tversky and Kahneman, (1974), which posits that, when uncertain, people make decisions/responses using an initial estimation, an anchor that they then adjust to correct for errors. Interestingly, these anchors are often based on egocentric representations (Epley et al., 2004; Gilovich et al., 2000; Keysar et al., 2000). For example, people often use their own experience as an anchor when estimating how their actions affect others (Gilovich et al., 2000) and when making judgements about how others perceive ambiguous stimuli (Epley et al., 2004). In the current task, participants may have used the original egocentric relation of self to the object as an anchor, which would result in dragging the object with them following a perspective shift. Adjustments are then made, considering the available information about the perspective shift (i.e., changes in the position of other features in the environment). However, if participants are uncertain about the exact nature of the perspective shifts, these adjustments are not sufficient, resulting in estimates that are biased towards the anchor (Quattrone, 1982; Tversky & Kahneman, 1974). This leads to a systematic shift in object position estimates in the direction of the perspective shift giving rise to the perspective shift-related bias.

An alternative explanation for the perspective shift-related bias relates to the specifics of the camera movement during the perspective shift. In our study, the camera moved on a circle such that a perspective shift to the left was realized by a camera translation to the left and a camera rotation to the right in order for the camera to remain directed towards the same point in the room. Such camera movements are typically used in spatial perspective taking tasks (Hilton et al., 2020; Montefinese et al., 2015; Muffato et al., 2019; Segen et al., 2021a, 2021b; Sulpizio et al., 2013). This combination of camera translation and rotation is chosen to ensure that the same part of the scene is visible in the images before and after the perspective shift. However, it produces images that can look surprisingly similar, and, as a result, may cause participants to underestimate the extent of the perspective shift. Underestimation of the perspective shift may lead participants to think that the camera movement was smaller than it was, yielding a bias in responses to the direction of the perspective shift. While in the current study we cannot distinguish between this explanation and the anchoring heuristic, we recently ran a follow-up experiment in which we systematically manipulated the way the camera moved during a perspective shift (Segen et al., 2021d). Results from this follow-up experiment provides support for the anchoring hypothesis and suggests that the influence of camera rotations is marginal.

A secondary aim of this study was to investigate if the bias in object position estimates results from systematic distortions in spatial memory. Importantly, we did not find a difference in the perspective shift-related bias between the *memory* and *perception* condition, suggesting that the systematic bias in errors in the direction of the perspective shift is not introduced by memory. This finding contrasts with previous research showing that biases in object location estimations are typically introduced by post-encoding processes (Crawford et al., 2016). For example, when participants estimate city locations from memory, they incorrectly place Montreal farther north than Seattle, influenced by their prior knowledge of Canada being to the north of the U.S (Friedman et al., 2005). In general, biases in object-location memory are typically explained by a postencoding Bayesian combination of more uncertain fine-grained information with the more certain category knowledge (Huttenlocher et al., 1991).

Yet, given our interpretation that the systematic bias is driven by processes underlying the perception/understanding of the perspective shift, it is not entirely surprising that we do not find differences between the memory and perception conditions. It should be noted that participants needed to engage in spatial perspective taking in both situations, with the only difference being that in the memory condition they needed to rely on a stored representation which they could either manipulate to match the test viewpoint or use as a reference to which the test stimuli viewpoint is matched.

To further investigate the role of memory in object location estimation we focused on the positions of the objects in the environment, as object location memory has been shown to be biased towards category prototypes (i.e., center of the screen, center of the quadrant; Crawford et al., 2016; Huttenlocher et al., 1991). Consistent with the prominent models of object location memory, such as the category adjustment model (Huttenlocher et al., 1991) and the dynamic field theory (Simmering et al., 2006; Spencer & Hund, 2002), we found that for the most leftward and rightward object positions, errors shifted away from the extremes towards the center. However, we did not find a systematic shift away from the central positions towards category prototypes that would be expected based on these models. This is consistent with our findings that the systematic bias is not introduced by memory, as the bias towards a prototype is a phenomenon that relates specifically to object-location memory and increases with memory delay. Instead, it is likely that the bias away from the extremes for the left and right clusters is a consequence of those objects appearing close to the ends of the scales, where they elicited a different response. For instance, in our anchoring and adjustment explanation of the perspective shift-related bias, adjustment processes would be impacted at the extremes since participants could not adjust beyond these endpoints. Notably, we did find a slight shift in participants’ errors to the right for the more central positions. A possible explanation for this bias is that the cameras were always directed towards the same spot in the environment that was slightly to the left of the center. If participants did not perceive this slight rotation and assumed that the camera faced the center of the room, they may have (mis)remembered the object to be slightly to the right. However, even if this was the case, the effect is very minor and overall, our results point to a systematic bias away from the extremes rather than towards a specific prototype with performance mainly influenced by the perception/understanding of the perspective shift rather than distortions introduced in memory.

Lastly, we turn our discussion to the relationship between the current findings of the perspective shift-related bias and the Reversed Congruency Effect, which manifested itself in better performance in estimating object movements that are in the opposite direction to the perspective shift and misjudgement of smaller movements in the same direction as the perspective, that we found in our previous study (Segen et al., 2021c). The unexpected finding of the Reversed Congruency Effect was an important motivator for the current study as it was the first report of a systematic bias related to the direction of the perspective shift. We proposed that the Reversed Congruency Effect was driven by the perspective shift-related bias. Specifically, if participants estimated the original object position as shifted in the direction of the perspective shift, as results from this study show, movement of an object in the opposite direction to the perspective shift would be perceived as larger and thus detected more easily. However, when the object moves in the direction of the perspective shift, the actual movement is attenuated by the expectation that the initial object position is “shifted” in the same direction. In such situations, smaller object movements may give rise to the impression of the object having moved in the opposite direction, as the expectation of original object position following a perspective shift may be shifted more in the direction of the perspective shift than the actual object movement.

To conclude, the current study shows that participants make systematic errors in the same direction as the perspective shift when estimating object locations across different perspectives. This perspective shift-related bias is present even in a perceptual version of the task and is likely driven by difficulties in understanding/perceiving the perspective shifts. We believe that the egocentric spatial relation between observer and target object acts as an anchor that participants fail to adequately adjust after the perspective shift. As a result, they make responses that are biased in the direction of the perspective shift. However, more research is needed to fully understand the mechanisms that give rise to the perspective shift driven bias in object location estimation. Importantly, the current findings are a conceptual replication of the Reversed Congruency Effect we reported in our previous study (Segen et al., 2021c). Further, the perspective shift-related bias was replicated in an online study (Segen et al., 2021e). The presence of the perspective shift-related bias across two different experimental paradigms (different sizes of perspective shifts, different tasks [determine direction of object movement vs estimate object positions]) and different experimental setting [lab vs online]) suggests that this is a robust effect that may translate to other studies that rely on static stimuli and perspective shifts. Thus, it is important for researchers who use similar paradigms to be mindful of this bias as it can greatly influence the interpretation of their results.

## Supplementary Information

ESM 1 (DOCX 23 kb)

## Footnotes

1 Even for more conservative effect sizes (5 and 7cm), the current sample size yielded >80% statistical power.
