# Supplementary material for: The role of memory and perspective shifts in systematic biases during object location estimation
Source: Atten Percept Psychophys. 2022 Feb 16;84(4):1208–19. doi: 10.3758/s13414-022-02445-y (PMC9076711; doi:10.3758/s13414-022-02445-y)
Supplement: Supplementary file 1 — (DOCX 23 kb) [file 13414_2022_2445_MOESM1_ESM.docx]

## Supplementary Materials

### Absolute error analysis

Absolute error was computed by calculating the distance on the horizontal plane between the correct position and the position selected by the participant. Given the predefined arrangement of positional markers that participants used to give a response the minimum error could be 14 cm (unless participants select the correct position) and maximum error depended on the position of the object during encoding. Interestingly, the results show that the absolute error was higher in the *Perception* compared to the *Memory* condition (β=3.002, SE=1.052, t=2.854) and there were no main effects of Cluster or PSD. An interaction was found between Condition and Cluster, such that in the *Memory* condition error in the *Left* cluster was lower than in the *Perception* condition (β=1.862, SE=0.393, t=4.736). No reliable differences between conditions was found for any of the other clusters.  In addition, we found an interaction between Cluster and PSD, with higher error in the *Right* cluster (β=3.271, SE=0.894, t=3.658) and lower error in the *Left* cluster (β=-2.459, SE=0.895, t=-2.747) when the PSD was to the *Left*. This suggests that the error increased when perspective shifts resulted in movements away from the original object position. This effect was amplified in the *Perception* compared to the *Memory* condition, with an even greater increase in error in the *Right*  (β=0.813, SE=0.393, t=2.068) and *Mid-right* (β=1.529, SE=0.395, t=3.870) cluster when the perspective shifted to the *Left* in the *Perception* condition and a greater decrease in error in the *Center-Left* (β=-0.969, SE=0.393, t=-2.463) and *Mid-Left* (β=-1.936, SE=0.394, t=-4.918) clusters.

Table 1 Coefficients from Absolute Error (cm) LMM analysis

|  | **Absolute Error** | | |
| --- | --- | --- | --- |
| *Predictors* | *Estimates* | *std. Error* | *t-value* |
| (Intercept) | 36.076 | 1.112 | **32.452** |
| Condition (*Perception*) | 3.002 | 1.052 | **2.854** |
| Cluster (*Right*) | -0.661 | 0.894 | -0.739 |
| Cluster (*Mid-right*) | 0.038 | 0.895 | 0.043 |
| Cluster (*Centre-left*) | 0.087 | 0.895 | 0.097 |
| Cluster (*Left*) | 0.327 | 0.895 | 0.365 |
| Cluster(*Mid-left*) | 0.089 | 0.895 | 0.099 |
| PSD (*Left*) | -0.391 | 0.573 | -0.682 |
| Condition (*Perception*)* Cluster (*Right*) | -0.632 | 0.393 | -1.608 |
| Condition (*Perception*)* Cluster (*Mid-right*) | 0.209 | 0.395 | 0.530 |
| Condition (*Perception*)* Cluster (*Centre-left*) | -0.244 | 0.393 | -0.620 |
| Condition (*Perception*)*Cluster (*Left*) | 1.862 | 0.393 | **4.736** |
| Condition (*Perception*)* Cluster (*Mid-left*) | 0.105 | 0.394 | 0.266 |
| Condition (*Perception*)* PSD(*Left*) | -0.486 | 0.446 | -1.089 |
| Cluster (*Right*)*PSD (*Left*) | 3.271 | 0.894 | **3.658** |
| Cluster (*Mid-right*)*PSD (*Left*) | 1.401 | 0.895 | 1.565 |
| Cluster (*Centre-left*)*PSD(*Left*) | 0.413 | 0.895 | 0.462 |
| Cluster (*Left*)*PSD (*Left*) | -2.459 | 0.895 | **-2.747** |
| Cluster (*Mid-left*)*PSD(*Left*) | -0.452 | 0.895 | -0.505 |
| Condition (*Perception*)* Cluster (*Right*)*PSD(*Left*) | 0.813 | 0.393 | **2.068** |
| Condition (*Perception*)* Cluster (*Mid-right*)*PSD (*Left*) | 1.529 | 0.395 | **3.870** |
| Condition (*Perception*)* Cluster (*Centre-left*)*PSD (*Left*) | -0.969 | 0.393 | **-2.463** |
| Condition (*Perception*)* Cluster (*Left*)*PSD (*Left*) | -0.399 | 0.393 | -1.015 |
| Condition (*Perception)**Cluster(*Mid-left*)*PSD(*Left*) | -1.936 | 0.394 | **-4.918** |

### Directional Error Analysis

Directional errors were used to investigate if Condition, Start Position and Perspective Shift Direction (PSD) have an effect on the direction of the errors. Negative errors indicate errors to the left and positive errors are errors to the left.

Table 2 Coefficients from Directional Error (cm) LMM analysis

|  | **Directional Error (cm)** | | |
| --- | --- | --- | --- |
| *Predictors* | *Estimates* | *std. Error* | *t-value* |
| (Intercept) | 3.355 | 1.480 | **2.267** |
| Condition (*Memory-Perception*) | 0.815 | 1.181 | 0.690 |
| Cluster (*Right*) | -15.594 | 2.081 | **-7.493** |
| Cluster (*Mid-right*) | -4.406 | 2.082 | **-2.116** |
| Cluster(*Centre-left*) | 0.111 | 2.082 | 0.053 |
| Cluster(*Left*) | 13.861 | 2.082 | **6.658** |
| Cluster(*Mid-left*) | 3.140 | 2.082 | 1.508 |
| PSD (*Right*-*Left*) | -10.944 | 1.796 | **-6.093** |
| Condition (*Memory*-*Perception*)* Cluster (*Right*) | 0.748 | 0.595 | 1.257 |
| Condition (*Memory-* *Perception*)* Cluster (*Mid-right*) | -3.206 | 0.598 | **-5.360** |
| Condition (*Memory-* *Perception*)* Cluster(*Centre-left*) | -0.269 | 0.596 | -0.452 |
| Condition (*Memory-Perception*)* Cluster(*Left*) | -0.091 | 0.595 | -0.153 |
| Condition (*Memory-Perception*)* Cluster(*Mid-left*) | 4.614 | 0.596 | **7.741** |
| Condition (*Memory-Perception*)* PSD (*Right*-*Left*) | 0.569 | 1.559 | 0.365 |
| Cluster (*Right*)*PSD (*Right*-*Left*) | 3.942 | 2.081 | 1.894 |
| Cluster (*Mid-right*)*PSD (*Right*-*Left*) | -1.412 | 2.082 | -0.678 |
| Cluster(*Centre-left*)*PSD (*Right*-*Left*) | -2.342 | 2.082 | -1.125 |
| Cluster(*Left*)*PSD (*Right*-*Left*) | -5.245 | 2.082 | **-2.519** |
| Cluster(*Mid-left*)*PSD (*Right*-*Left*) | -1.875 | 2.082 | -0.901 |
| Condition (*Memory-Perception*)* Cluster (*Right*)*PSD (*Right-Left*) | -0.897 | 0.595 | -1.508 |
| Condition (*Memory-Perception*)* Cluster (*Mid-right*)*PSD (*Right-Left*) | 1.434 | 0.598 | **2.397** |
| Condition (*Memory-Perception*)* Cluster(*Centre-left*)*PSD (*Right-Left*) | -0.629 | 0.596 | -1.056 |
| Condition (*Memory-Perception*)* Cluster(*Left*)*PSD (*Right-Left*) | -0.354 | 0.595 | -0.594 |
| Condition (*Memory-Perception)**Cluster(*Mid-left*)* PSD (*Right-Left*) | -1.436 | 0.596 | **-2.409** |

**Re-analysis of Signed and Directional Errors without the extreme object clusters**

|  | **Signed Error** | | |
| --- | --- | --- | --- |
| *Predictors* | *Estimates* | *std. Error* | *t-value* |
| (Intercept) | 13.452 | 2.074 | 6.487 |
| Condition (*Memory*-*Perception*) | -0.859 | 1.581 | -0.543 |

|  | **Directional Error** | | |
| --- | --- | --- | --- |
| *Predictors* | *Estimates* | *std. Error* | *t-value* |
| (Intercept) | 4.069 | 1.790 | 2.274 |
| Condition (*Memory*-*Perception*) | 0.744 | 1.264 | 0.588 |
| Cluster (*Mid-right*) | -5.153 | 2.255 | -2.285 |
| Cluster(*Centre-left*) | -0.087 | 2.255 | -0.038 |
| Cluster(*Mid-left*) | 2.718 | 2.255 | 1.206 |
| PSD (*Right*-*Left*) | -13.478 | 2.047 | -6.583 |
| Condition (*Memory*-*Perception*) * Cluster (*Mid-right*) | -2.814 | 0.523 | -5.384 |
| Condition (*Memory*-*Perception*) * Cluster(*Centre-left*) | -0.481 | 0.521 | -0.923 |
| Condition (*Memory*-*Perception*) * Cluster(*Mid-left*) | 4.442 | 0.522 | 8.515 |
| Condition (*Memory*-*Perception*) *PSD (*Right*-*Left*) | 0.860 | 1.609 | 0.535 |
| Cluster (*Mid-right*) *PSD (*Right*-*Left*) | 1.153 | 2.255 | 0.511 |
| Cluster(*Centre-left*) *PSD (*Right*-*Left*) | -0.261 | 2.255 | -0.116 |
| Cluster(*Mid-left*) *PSD (*Right*-*Left*) | 0.440 | 2.255 | 0.195 |
| Condition (*Memory*-*Perception*) * Cluster (*Mid-right*) *PSD (*Right*-*Left*) | 0.815 | 0.523 | 1.560 |
| Condition (*Memory*-*Perception*) * Cluster(*Centre-left*) *PSD (*Right*-*Left*) | -0.521 | 0.521 | -1.000 |
| Condition (*Memory*-*Perception*) * Cluster(*Mid-left*) *PSD (*Right*-*Left*) | -1.394 | 0.522 | -2.673 |
